# Gender and Regional Differences in Sleep Quality and Insomnia: A General Population-based Study in Hunan Province of China

**DOI:** 10.1038/srep43690

**Published:** 2017-03-06

**Authors:** Jinsong Tang, Yanhui Liao, Brian C. Kelly, Liqin Xie, Yu-Tao Xiang, Chang Qi, Chen Pan, Wei Hao, Tieqiao Liu, Fengyu Zhang, Xiaogang Chen

**Affiliations:** 1 Department of Psychiatry & Mental Health Institute of the Second Xiangya Hospital, Central South University. National Clinical Research Center on Mental Disorders & National Technology Institute on Mental Disorders. Hunan Key Laboratory of Psychiatry and Mental Health, 139 Renmin (M) Rd, Changsha, Hunan 410011, P. R. China; 2 Department of Psychiatry and Biobehavioral Sciences, UCLA, 760 Westwood Plaza, Los Angeles, CA 90095, USA; 3 Department of Sociology & Center for Research on Young People’s Health (CRYPH), Purdue University, 700 W State Street, West Lafayette, IN 47907, USA; 4 Changsha Social Work College, 22 Xiangzhang Rd, Yuhua, Changsha, Hunan, 410116, P. R. China; 5 Unit of Psychiatry, Faculty of Health Sciences, University of Macau, Avenida da Universidade, 3/F, Building E12, Macau SAR, Taipa, P. R. China; 6 Clinical Psychology Department, the Third Xiangya Hospital, Central South University, Changsha, Hunan 410013, China

## Abstract

Insomnia and the inability to sleep affect people’s health and well-being. However, its systematic estimates of prevalence and distribution in the general population in China are still lacking. A population-based cluster sampling survey was conducted in the rural and urban areas of Hunan, China. Subjects (*n* = 26,851) were sampled from the general population, with a follow-up using the Pittsburgh Sleep Quality Index (PSQI) for interview to assess quality of sleep and Insomnia (PSQI score >5). While the overall prevalence of insomnia was 26.6%, and little difference was found between males (26.3%) and females (27.0%); the mean PSQI score was 4.26 (±2.67), and significant higher in females (4.32 ± 2.70) than males (4.21 ± 2.64, p = 0.003). Individuals in the rural areas tended to report a higher PSQI score (4.45 ± 2.81) than urban residents did (4.18 ± 2.60) (p < 0.001) and the estimates of prevalence of insomnia was 29.4% in the rural areas, significant higher than 25.5% in the urban areas (p < 0.001). Multiple logistic regression analysis showed that female gender, older age, higher level of education, being unmarried, living in the rural area, cigarette smoking and alcohol drinking were associated with insomnia. Our study may provide important information for general and mental health research.

Sleep is an important physiological process that affects health and well-being and the experience of proper sleep varies across individuals. Sleep quality is a measure of the feeling that a person would have of being energetic, active, and ready for a new day. It includes quantitative aspects such as sleep duration, sleep latency, and number of arousals, as well as qualitative aspects such as the depth and feeling of restfulness upon awakening^1^. While the direct benefits of high-quality sleep are not well quantified across populations, it is understood that sleep loss or insomnia is a serious public concern that may affect quality of life and well-being^2^. Epidemiological surveys indicate that about 15% to 35% of adult individuals have frequent sleep disturbances, including difficulty falling asleep or maintaining sleep^1^.

Poor sleep quality may have serious health and personal consequences. It can lead to impairment or poor performance during daytime school or work, increase risk for motor vehicle or occupational accidents, exacerbate medical and psychiatric conditions, and result in diminished quality of life^3^. Frequent difficulties initiating sleep have been associated with higher mortality among men but not women^4^. Thus, sleep disturbances affect a wide range of aspects of health and well-being.

The measurement and assessment of sleep disturbances is particularly important with respect to study of sleep and its related effects. Proper sleep may not be completely assessed by a single dimension such as duration, but have to be considered as a multi-dimensional construct. Such measurement considerations can influence both prevalence estimates and examining the association of sleep disturbances with health outcomes. The most widely used standardized measure is the Pittsburgh Sleep Quality Index (PSQI), which is designed to differentiate between “good” sleepers and “poor” sleepers and to identify subgroups of poor sleepers^1^. As such, it permits a comprehensive assessment of sleep.

There has been lack of consistency in the studies of gender differences in sleep quality. Buysse *et al*. found an association of female gender with high PSQI scores in a community-dwelling adults sample^5^. A recent school-based study with 7,507 children and adolescents showed higher prevalence of insomnia symptoms in girls than boys^6^, indicating that a gender difference may extend across the life course. However, Buysse and colleagues previously found no significant gender differences in PSQI among healthy young and elderly subjects^7^. When it comes to clinical patients, there was a significant difference in mean PSQI scores between male and female patients in the last week of study and in the pre-illness period^8^. However, men may consider their quality of sleep better than women^9^. Nevertheless, considering a line of evidence on morphologic differences between males and females in circadian clock genes, respiratory control, the action of sex hormones, stress responses on sleep mechanisms, and social patterning of behaviors that affect sleep, the difference in sleep quality between genders is likely real^3^,^10^.

Quality of sleep may vary substantially by gender and residential location. A great attention has been paid to social, demographic, and societal influences on sleep^11^,^12^,^13^. Previous research identified that neighborhood environments may influence sleep-related outcomes^14^. An examination of residential status is particularly important in China as the residential registration system (*hukou*) has restricted the mobility of residents over the past decades. Residential location was indicated as an important factor associated with sleep quality in the rural and urban areas near Beijing and Shanghai^15^ and in the United States^16^. However, a robust population-based estimates of sleep disturbances and insomnia are scarce in the general populations of mainland China; and little had been known about the differences in sleep quality by gender and residential location.

The main purpose of this study is to evaluate differences in sleep quality and insomnia by gender and residential location. We also tried to assess association of factors such as age, marital status, educational level, cigarette smoking and alcohol drinking with rate of insomnia (with PSQI > 5) in the general population sample. Our primary hypotheses were: (1) the mean PSQI scores and the prevalence of insomnia will be higher for women, and (2) poor sleep quality and prevalence of insomnia will be higher in urban areas given the increased exposure to noise and light pollution as well as the more intense pace of life in such locales.

## Results

### Participant demographic characteristics

Among the total 26,851 participants, 26,766 participants (14,215 men and 12,551 women) were included in demographic analysis. The mean age was 38.1 (±16.05) years old, with 42.8 (±17.53) in the rural residents, which was significantly older than 36.0 (±14.85) in the urban residents (p < 0.001). Compared with female participants, males tended to have a higher level of education, Body Mass Index (BMI), personal income, as well as were more likely to be employed, married, urban residents, and overwhelmingly more likely to smoke cigarettes and drink alcohol (Table 1).

### Sleep quality and poor sleeping

After the exclusion of individuals with missing information on demographics and PSQI, 25,827 participants were included in the final analysis of sleep quality and poor sleeping. Analysis of the PSQI score estimated that the mean sleep latency (defined as the amount of time it takes to fall asleep after lights off) was 21.05 (±19.33) minutes (20.48 ± 19.99 in males and 21.05.8 ± 18.57 in females); the mean of total sleep time was 454.75 (±77.30) minutes (451.61 ± 78.38 in males and 458.39 ± 75.94 in females). Detailed statistics of PSQI components and total score in the overall sample and by gender are presented in Table 2.

Female participants tended to have higher scores than males in multiple PSQI component scores. We observed significant difference in PSQI components of subjective sleep quality, sleep latency, sleep duration, habitual sleep efficiency, and sleep disturbance between men and women (p < 0.0001). However, there were no significant gender differences in other two PSQI components - need for sleep medications (p = 0.437) and daytime dysfunction (p = 0.220). While females had a higher PSQI total score than males (p = 0.003), there was no significant difference (p = 0.232) when it came to the rate of poor sleepers between males (26.3%) and females (27.0%). It is noteworthy that the lowest PSQI component score was found with “a need for sleep medications” in overall participants (0.11 ± 0.38), with less than 10% of participants reporting a need for sleep medications during the past month before the time of survey (6.9% reported less than once a week, 1.4% reported once or twice a week and 0.4% reported ≥3 times a week (Table 2).

### PSQI total score and prevalence of insomnia by gender and region

Considering that there was a significant difference in PSQI total score between males and females, we performed a stratification analysis by region, as urban residence is highly heterogeneous in the size of city, which may have different implications for the sleep quality (Table 3). Overall, there was a significant difference in PSQI total score between the rural and urban areas (4.45 ± 2.81 vs 4.18 ± 2.60, p < 0.01). However, when examining the urban sample alone, we found that the difference in PSQI total score between men and women were less or not significant in individuals who lived in small town (p = 0.059), county city (p = 0.048), prefecture city (p = 0.0894), all of which were small to medium size city; but we still observed a significant gender difference in people who lived in Changsha, the largest metropolitan and the capital city of Hunan (p = 0.02).

Similar stratification analysis of prevalence of insomnia was performed and showed no significant differences in insomania by gender and by region (p > 0.11). However, we found a significant difference (p < 0.001) in prevalence of insomnia between urban (25.5%) and rural areas (29.4%). Individuals who lived in the rural areas had a high likelihood of poor sleeping (Table 4).

### Factors associated with increased likelihood of sleep problems or insomnia

Based on descriptive analysis and previous literature, we examined the association of factors such as age, gender, marital status, level of education, rural and urban residence, cigarette smoking and alcohol drinking, with poor sleeping. Based on simple linear regression analysis, we found that all seven factors were associated with PSQI total score (sleep quality) (see Table 5). Further with multiple logistic regression analysis, we confirmed that more likelihood of insomnia was associated with female gender (OR = 1.367, P < 0.001), rural residence (OR = 1.115, p = 0.002), older age (OR = 1.026, p < 0.001), higher level of education (OR = 1.02, p < 0.001), unmarried status such as divorced, separated or widowed status (OR = 1.587, p < 0.001), cigarette smoking (OR = 1.468, P < 0.001) and alcohol drinking (OR = 1.265, p < 0.001) (Table 6). The effect size for some of these factors were not trival.

## Discussion

In this study, we provide the first estimates of sleep quality and insomnia by gender and residence and examination of associated factors in a large sample of the general population in mainland China. While reporting a similar rate of insomnia, women tended to have a poorer sleep as measured by PSQI total score; We also found a significant difference in poorer sleep quality and higher prevalence of insomnia in rural than urban residents, but inconsistent with other studies; and multiple regression analysis also showed a number of demographic variables are also associated with sleep quality.

Overall, insomnia is a common health problem in the general population of central China. According to our estimates, more than one fourth of individuals had insomnia during the past month at the time of survey. These findings are similar, with some variation due to study populations, to the prevalence of sleep problems found in epidemiological studies conducted in other parts of China. Previous studies showed that an overall point estimate of the prevalence of insomnia (PSQI score > 5) was 39.4% in the general population of Hong Kong^17^,^18^, and reported a 32.9% of insomnia among the elderly Chinese in Shandong^19^; Community- or population-based cross-sectional surveys also reported that the prevalence of poor sleep (PSQI score > 5) in older adults was 20.67% in Guangdong^20^, and 26.36% in adults aged 20 and above in the rural areas of Liaoning^21^. A Japanese nationwide survey in general population reported the overall prevalence of insomnia was 32.7%^22^; and A German study of community sample (aged 18–80 years) estimated that 36% of the general population had bad sleep quality (PSQI > 5)^23^. Our estimate of prevalence appears lower than those previous studies. While population structure may contribute to this difference, ethnicity^13^, socioeconomic status, and even geographic locations^11^ may affect sleep quality and insomnia. These are all warranted to have a further investigation of the determinants of sleep quality and insomnia.

There was a significant difference in sleep quality but not prevalence of insomnia between men and women. Consistently with previous studies^3^, our study indicates that women had poorer sleep quality than men. However, we found no significant difference in the overall prevalence of insomnia between men and women; this appears contradictory to other previous reports^22^,^24^. For example, the prevalence of having sleep problems were 26.4% in men and 31.1% in women in the general Japanese adult population^25^, and 9.5% in men and 14.3% in women in another sample from northern Japan^26^. Defined by PSQI > 5, another urban population-based sample in Iran showed 27% in men versus 35% in women^27^. The absence of a gender difference in the prevalence of insomnia in the central China merits a further investigation.

It was a little surprise to see a significant difference in poorer sleep quality and higher prevalence of insomnia in rural than urban residents. We had anticipated that the greater noise and light pollution as well as more fast-paced urban life may have diminished the likelihood of quality sleep. This finding is also contradictory to other studies. A cross-sectional survey conducted in older adults in the urban and rural areas of Beijing and Shanghai reported that, despite lower socio-economic status and poor medical insurance coverage, rural residents were more likely to report better sleep quality than urban residents^15^. Yet, a study of middle age people in Beijing found that while overall there was no significant difference between rural and urban residents in any type of insomnia, more rural (21.1%) than urban(14.9%) residents reported the highest rate of at least one type of insomnia (overall 17.1%)^28^.

There may be multiple explanations to the higher prevalence of insomnia in the rural areas that may deserve some attentions. In the rural areas of China, high demands for physical work often leads to musculoskeletal pain or illness that may affect sleep quality among rural workers^29^. We may also consider that the higher level of insomnia in the rural areas may be influenced by the massive rural-to-urban migration occurring in China. Rural migrants may suffer more from stressful events that may result from finding a job and decent place to live or more commutes between home and work place. The current study showed that rural participants was older than the urban participants, which may be due to the rural to urban migration of younger people^30^, leaving relatively older adults who may suffer from stress from separation with their migrant daughters and sons and in general may have more sleep problems as brain aging. Besides older age, limited access to quality health services may result in a higher prevalence of insomnia in rural areas^31^; and availability and quality of health services were different between urban and rural areas of China^32^,^33^. Future investigations should consider which social and institutional factors related to migration may cause the difference in insomnia in the general population between rural and urban locations.

Our multiple logistic regression model also showed that female gender, older age, high level of education, unmarried status, living in the rural areas, and cigarette smoking and alcohol drinking were associated with higher odds of insomnia. Those demographic variables have been established elsewhere in the literature. A telephone survey among the Hong Kong Chinese population also showed a higher prevalence of insomnia in females^34^. The gender differences in insomnia were confirmed by a meta-analysis^35^. Clinical and pre-clinical studies suggest that biological sex and sex steroids influence sleep behavior and sleep disorders^10^,^36^. A line of research has shown that insomnia was significantly associated with older age^22^,^24^,^37^,^38^; and marital status and level of education were associated with insomnia^28^. However, Xiang et al showed that low level of education (illiteracy and primary school) was significantly associated with insomnia^28^. One of the possible reasons we found different results may be due to the ever-increasing occupational stress among well-educated people in China.

Cigarette smoking and alcohol use were associated with sleep problems. Our previous studies showed frequently sleep problems among drug users^39^,^40^. The current study did find that cigarette smokers and alcohol drinkers had higher odds for poor quality of sleep, which also was consistent with other previous studies^41^,^42^,^43^. As use of substance in China is highly gendered, more so than in other regions of the world, it remains important to further examine the role of such substance use behaviors in the gender and regional differences described above. It remains interesting that men use substances much more so than women use, yet do not seem proportionally affected in their overall prevalence of insomnia. Further explication of the complex underlying mechanisms between insomnia and age, levels of education, marital status, and residential regions is needed.

This study has a number of strengths, including the large sample size, the high rate of participation, the use of well-established instruments to examine sleep problems, and the questionnaire-based door-to-door interview. However, there were some limitations. First, although this is a randomly selected sample of community-dwelling participants, we did not use a multistage, stratified sampling method to select a provincially representative sample of the general population. That may limit the generalizability of this study. Second, in the present study, sleep quality and insomnia were assessed using a retrospective, self-reported approach using PSQI, which may be subject to some reporting errors; participants with insomnia may be more likely to overestimate their sleep latency and underestimate their sleep duration. Although objective measures (i.e., physiologic measurements such as electroencephalography) are desirable, they have, however, not been regularly incorporated into such a large epidemiologic study of the general population. Thus, self-reports and interview-based measures remain the most widely used measures in community surveys^44^. Third, we were not able to distinguish between primary and secondary insomnia and to classify insomnia into DSM-5 subtypes, such as primary insomnia, insomnia related to a mental disorder, for example, substance-induced insomnia are very common^39^,^40^. In addition, because the analysis is cross-sectional, we are unable to make inferences of causal relationships for the factors identified, such as that between substance use and sleep quality. For example, insomnia is an important indicator of perceived physical and mental health status^22^. However, the focal point of gender within our study permits some assurance with respect to the directionality of such a causal pathway.

In conclusion, the present study, based on the PSQI, showed a high prevalence of insomnia in the general population of central China. Females reported poorer sleep quality than males, and rural participants reported poorer sleep quality and higher prevalence of insomnia than urban participants did. Older age, high levels of education, unmarried status, cigarette smoking and alcohol drinking were also associated with insomnia. Further studies using longitudinal designs are warranted to examine the community-level, psychosocial, and biologic factors of insomnia. Within the public health system, further attention has to be given to sleep quality and insomnia.

## Methods

### Design

A cross-sectional survey design was used to explore sleep quality. We used a community-based method to select a sample of persons 12 years of age or older in the general population from Hunan Province, a province located in the south-central region of China, with a population of 65.68 million (according to The Sixth National Population Census of the People’s Republic of China in 2010). Hunan has 14 prefecture-level regions, which are subdivided into 122 county-level administration divisions (including 34 districts, 16 county-level cities, 65 counties, and 7 autonomous counties).

### Study Participants

**Figure 1:**
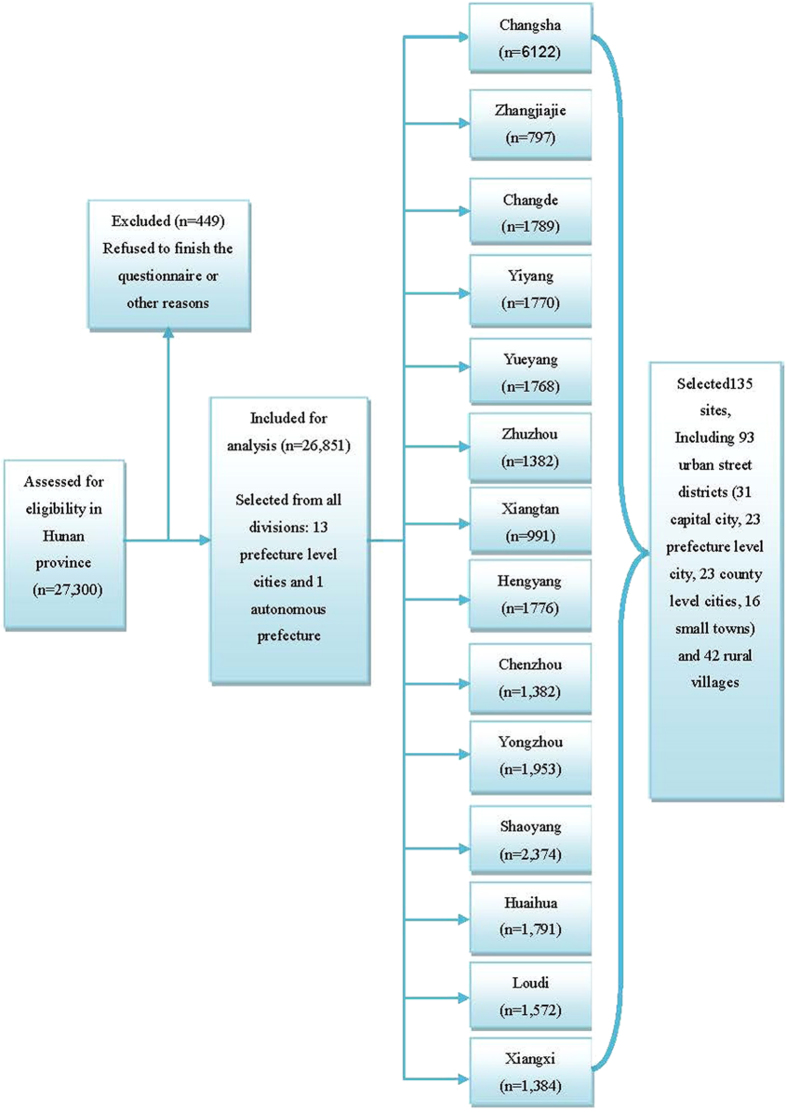
Flowchart of the sampling procedure in the Hunan province, China for sleeping quality study.

In this population-based cross-sectional survey, participants were recruited from September 2012 to October 2012 in Hunan province. We used a community-based method to select participants from all divisions (13 prefecture level cities and 1 autonomous prefecture) of Hunan province, including 93 urban street districts (31 capital cities, 23 prefecture level cities, 23 county level cities, 16 small towns) and 42 rural villages were randomly selected, which yielded 135 sites in total. In each prefecture level city or autonomous prefecture, at least 1 prefecture level city, 1 county level city, 1 small town and 1 rural village has been randomly selected. Only a person aged 12 years or older and living in their current residence for 5 years or longer were selected from each household without replacement. 27,300 people were randomly selected from the selected sites and invited to participate in the study; 26,851 persons completed the study (details showed in Fig. 1). The overall response rate was 98.4% (449 participants refused to respond, including 228 men and 221 women; 243 lived in urban areas and 206 lived in rural areas). After the exclusion of missing demographic and PSIQ information, 25,827 participants were included in the final analysis. The ages of the participants ranged from 12 to 99 years, with an average age of 38.1 ± 16.05 years.

The protocol was approved by the university ethics committee (The Second Xiangya Hospital of Central South University Review Board, No. S101, 2011) and the studies were carried out in accordance with the Declaration of Helsinki. Subjects were fully informed about the survey. All study participants gave verbal informed consent. Eligible subjects were 12 years of age or older who could fully understand the survey.

### Procedure

At each selected site, participants from door to door were invited to fill out the survey if they consented to participate. Every household member aged 12 or more years was encouraged to participate by a face-to-face interview. As part of the consent process, participants were provided with a detailed explanation of the objectives of the study and study expectations. Participants were advised of their ability to withdraw from the study at any point. Issues of confidentiality and anonymity were discussed. Verbal informed consent was obtained. Participants were encouraged to answer the questionnaire independently and as soon as possible. Those who were unable to recognize 1,500–2,000 Chinese characters were defined as illiterate. In such cases, the staff would read and explain each question, and helped illiterate participants to write down the answer if necessary.

### Assessment measures

In this face-to-face survey, each participant was asked to answer a self-reported battery of questionnaires that consisted of socio-demographic and sleep-related information. The demographic section gathered information on age, gender, marital status, employment status, and years of education, height, weight, personal income, cigarette smoking, and alcohol drinking. The Pittsburgh Sleep Quality Index (PSQI)^1^ was used to assess sleep. The PSQI is a validated, self-administered questionnaire used that measures sleep quality and disturbances over a one-month time interval. It is a seven-item questionnaire with each item rated from 0 to 3 (sleep duration, sleep efficiency, sleep latency, sleep disturbance, daytime dysfunction, frequency of sleep medications, and subjective sleep quality) with scores ranging between 0 and 21 points; higher scores indicates a lower quality of sleep. A PSQI global score greater than 5 indicates ‘poor sleep’ with a sensitivity of 89.6–98.7% and specificity of 84.4–86.5% and was used to define symptoms of insomnia in this study^1^,^45^. The questionnaire is easy to handle and can be completed within 5 minutes. The Chinese version of PSQI has been extensively used in patients and general populations with good reliability and validity^39^,^46^.

### Statistical analysis

Statistical analysis was performed using SPSS (Version 16, SPSS Inc., Chicago, IL, USA). To address missing data, incomplete questionnaires missing the variables of interest were excluded. Descriptive statistics were examined on demographic characteristics and pooled responses, including the mean, standard deviation for continuous variables, and percentage for categorical variables. Between-group comparisons were performed using χ^2^ (Chi-square) for categorical variables and the Wilcoxon rank sum test (Mann-Whitney U-test) (as the data are not perfect normal distributions) for continuous measures. The subjects were classified into two groups by sleep quality: good if PSQI score ≤ 5; poor sleepers or insomnia if PSQI score > 5. Simple linear regression analysis was used to examine the association between continuous variables (PSQI total score) and each of the associated factors (age, gender, marital status, levels of education, rural or urban residence, with or without cigarette smoking, and with or without alcohol drinking). Then, multiple logistic regression analysis was performed to obtain the odds ratios between the associated factors and poor sleep. A power analysis was also performed, and the odds ratios (OR) and 95% confidence intervals (CI) of the predictors were derived. A p-value threshold of 0.05 was set to determine statistical significance.

## Additional Information

**How to cite this article**: Tang, J. *et al*. Gender and Regional Differences in Sleep Quality and Insomnia: A General Population-based Study in Hunan Province of China. *Sci. Rep.* **7**, 43690; doi: 10.1038/srep43690 (2017).

**Publisher's note:** Springer Nature remains neutral with regard to jurisdictional claims in published maps and institutional affiliations.

## Figures and Tables

**Table 1:** Demographic characteristics in overall participants and by gender.

Variables	Overall (n = 26,766)	Male (n = 14,215)	Female (n = 12,551)	p*
**Age** (**yrs**), **M ± SD**	38.1 ± 16.05	37.8 ± 16.52	38.4 ± 15.49	0.003
**Education** (**yrs**), **M ± SD**	10.6 ± 3.79	10.9 ± 3.69	10.3 ± 3.87	<0.001
1. Illiteracy or primary school, %	19.00	16.30	22.00	—
2. Middle school, %	32.50	31.40	33.80	—
3. High school, %	23.30	25.50	20.90	—
4. University, %	25.20	26.80	23.30	—
**BMI**^x^, **M ± SD**	21.2 ± 2.92	21.5 ± 2.88	20.8 ± 2.91	<0.001
**Personal income** (**CNY**)	2231 ± 3392.81	2581 ± 3901.18	1835.9 ± 2649.66	<0.001
**Employed %**	78.10	79.30%	76.80	<0.001
**Female %**	46.90	—	—	—
**Married %**	67.70	64	72.10	<0.001
1. First marriage, %	67.10	63.30	71.60	—
2. Re-married, %	0.60	0.70	0.50	—
**Unmarried %**	32.30	36.00	27.90	<0.001
1. Never married, %	26.60	30.70	21.80	—
2. Divorced, %	2.00	1.90	2.00	—
3. Widowed, %	3.20	2.90	3.60	—
4. Separated, %	0.50	0.60	0.50	—
**Rural region%**	31.20	30.20	32.20	<0.001
**Urban region %**	68.80	69.80	67.80	<0.001
1. Provincial capital city (Changsha), %	22.80	23.50	22.50	—
2. Prefecture-level city, %	16.30	16.20	16.10	—
3. County-level city, %	17.10	17.30	16.80	—
4. Small town, %	12.60	12.80	12.40	—
**Cigarette smoker%**	25.30	45	3	<0.001
**Smoked cigarettes**/**day**	18.4 ± 14.84	18.3 ± 14.80	18.7 ± 15.46	0.716
**Alcohol drinker%**	20	33.80	4.20	<0.001

M: mean; SD: standard deviation; n: number; %: the percentage of subjects; Cigarette smoker, defined as smoked more than 100 cigarettes in the life time; Alcohol drinking, defined as drunk no less than 30 g alcohol (equal to 900 ml beer) per week.

^*^P value, test for the difference by gender.

^x^Body Mass Index (BMI), calculated as weight (in kilogram) divided by height (in meter) squared.

**Table 2:** PSQI component scores and total score in all participants and by gender.

Variables	Total (n = 25,827)	Male (n = 13,674)	Female (n = 12,153)	P*
**Subjective sleep quality**, **M ± SD**	0.82 ± 0.65	0.8 ± 0.65	0.84 ± 0.65	<0.001
**Sleep latency**, **M ± SD**	0.77 ± 0.73	0.74 ± 0.72	0.80 ± 0.74	<0.001
≤15 min, %	38.60	40.50	36.30	—
16–30 min, %	48.20	47.00	49.50	—
31–60 min, %	11.00	10.40	11.60	—
>60 min, %	2.30	2.00	2.50	—
**Sleep duration**, **M ± SD**	0.58 ± 0.78	0.62 ± 0.79	0.54 ± 0.76	<0.001
>7 h, %	58.10	55.90	60.60	—
5–7 h, %	40.40	42.50	38.10	—
<5 h, %	1.50	1.60	1.30	—
**Sleep time** (**minute**)	454.75 ± 77.30	451.61 ± 78.38	458.39 ± 75.94	<0.001
Habitual sleep efficiency*, **M ± SD**	0.33 ± 0.73	0.32 ± 0.72	0.35 ± 0.73	<0.001
≥85%, %	78.60	79.70	77.30	—
75–84%, %	13.30	12.40	14.30	—
65–74%, %	4.60	4.30	4.80	—
<65%, %	3.60	3.50	3.60	—
**Sleep disturbance**, **M ± SD**	0.77 ± 0.55	0.76 ± 0.55	0.79 ± 0.55	<0.001
**Need for sleep medications**, **M ± SD**	0.11 ± 0.38	0.11 ± 0.38	0.11 ± 0.39	0.437
**Not during the past month**, **%**	91.30	91.40	91.20	—
**Less than once a week**, **%**	6.90	6.70	7.10	—
**Once or twice a week**, **%**	1.40	1.50	1.30	—
≥**3 times a week**, **%**	0.40	0.40	0.40	—
**Daytime dysfunction**, **M ± SD**	0.88 ± 0.84	0.88 ± 0.84	0.89 ± 0.83	0.220
**PSQI total score**, **M ± SD**	4.26 ± 2.67	4.21 ± 2.64	4.32 ± 2.70	0.003
**Insomnia** (**PSQI > 5**), **%**	26.60	26.30	27.00	0.232

M: mean; SD: standard deviation; n: number; %: the percentage of subjects. PSQI: Pittsburgh Sleep Quality Index.

^*^P value, test for the difference by gender.

Score ranges from 0 to 3, with higher scores indicating poorer functioning. Habitual sleep efficiency* = total hours of sleep/(get-up time−bedtime) × 100%.

Good sleepers was defined as PSQI < 5.

**Table 3:** PSQI total score (M ± SD) by gender and residence location.

	Overall	male	female	p*
**Overall**	4.26 ± 2.67	4.21 ± 2.64	4.32 ± 2.70	0.003
**Rural**^**x**^	4.45 ± 2.81	4.41 ± 2.79	4.49 ± 2.84	0.209
**Urban**^**x**^ (**overall**)	4.18 ± 2.60	4.12 ± 2.58	4.25 ± 2.62	0.003
Small town	4.16 ± 2.59	4.07 ± 2.54	4.28 ± 2.64	0.059
County-level city	4.18 ± 2.68	4.11 ± 2.66	4.27 ± 2.70	0.048
Prefecture-level city	4.06 ± 2.61	4.04 ± 2.55	4.07 ± 2.66	0.894
Provincial capital city	4.28 ± 2.54	4.22 ± 2.56	4.35 ± 2.51	0.020

M: mean; SD: standard deviation; PSQI: Pittsburgh Sleep Quality Index.

^*^P value for difference in PSQI score by gender.

^**x**^P < 0.01, testing for difference in PSQI total score by region (Mann-Whitney U-test).

**Table 4:** Prevalence of insomnia (%) by region and gender.

	Overall	male	female	P*
**All regions**, **%**	26.60	26.30	27.00	0.232
**A**. **Rural**^x^, **%**	29.40	28.90	29.80	0.366
**B**. **Urban**^x^ (**overall**), **%**	25.50	25.30	25.80	0.618
B.1. Small town, %	24.80	24.20	25.60	0.394
B.2. County-level city, %	25.90	25.70	26.10	0.754
B.3. Prefecture-level city, %	23.50	23.70	23.30	0.771
B.4. Provincial capital city, %	27.00	26.50	27.50	0.389

M: mean; SD: standard deviation; n: number; %: proportion of insomnia.

PSQI: Pittsburgh Sleep Quality Index.

^*^P value, testing for gender difference in insomnia by region from Chi-square test.

^x^Significant difference in proportion of poor sleepers in overall sample between rural and urban areas (p < 0.001, Chi-square test).

**Table 5:** Simple linear regression analysis of PSQI total score.

Variable	B	p*
Gender(female = 1, male = 0)	0.020	0.001
Age(yrs)	0.210	<0.001
Year of education	−0.037	<0.001
Marriage (Married = 1, others = 0)	0.023	<0.001
Residency (urban = 1, rural = 0)	−0.046	<0.001
Cigarette smoking(smoking = 1, nonsmoking = 0)	0.100	<0.001
Alcohol drinking(alcohol use = 1, no alcohol = 0)	0.086	<0.001

B: standardized regression coefficient (Beta). PSQI: Pittsburgh Sleep Quality Index.

^*^p value for association.

**Table 6:** Multiple logistic regression model predicting insomnia.

Variable	B	OR	CI	p*
Female Gender	0.313	1.367	(1.272, 1.469)	<0.001
Age (yrs)	0.025	1.026	(1.023, 1.028)	<0.001
Education (yrs)	0.020	1.02	(1.011, 1.029)	<0.001
Unmarried	0.468	1.597	(1.487, 1.716)	<0.001
Urban residence	−0.109	0.897	(0.836, 0.962)	0.002
Current cigarette smoking	0.384	1.468	(1.349, 1.596)	<0.001
Current alcohol drinking	0.235	1.265	(1.164, 1.375)	<0.001

OR: odds ratio. CI: 95% confidence intervals. PSQI: Pittsburgh Sleep Quality Index.

^*^p value for significant association.
